# Therapeutic Effects of Mesenchymal Stromal Cell-Derived Small Extracellular Vesicles in Oxygen-Induced Multi-Organ Disease: A Developmental Perspective

**DOI:** 10.3389/fcell.2021.647025

**Published:** 2021-03-16

**Authors:** Angeles Fernandez-Gonzalez, Gareth R. Willis, Vincent Yeung, Monica Reis, Xianlan Liu, S. Alex Mitsialis, Stella Kourembanas

**Affiliations:** ^1^Division of Newborn Medicine, Department of Pediatrics, Boston Children’s Hospital, Boston, MA, United States; ^2^Department of Pediatrics, Harvard Medical School, Boston, MA, United States

**Keywords:** development, BPD, preterm, oxygen, inflammation, multiorgan, MEx

## Abstract

Despite major advances in neonatal intensive care, infants born at extremely low birth weight still face an increased risk for chronic illness that may persist into adulthood. Pulmonary, retinal, and neurocognitive morbidities associated with preterm birth remain widespread despite interventions designed to minimize organ dysfunction. The design of therapeutic applications for preterm pathologies sharing common underlying triggers, such as fluctuations in oxygen supply or in the inflammatory state, requires alternative strategies that promote anti-inflammatory, pro-angiogenic, and trophic activities—ideally as a unitary treatment. Mesenchymal stem/stromal cell-derived extracellular vesicles (MEx) possess such inherent advantages, and they represent a most promising treatment candidate, as they have been shown to contribute to immunomodulation, homeostasis, and tissue regeneration. Current pre-clinical studies into the MEx mechanism of action are focusing on their restorative capability in the context of preterm birth-related pathologies, albeit not always with a multisystemic focus. This perspective will discuss the pathogenic mechanisms underlying the multisystemic lesions resulting from early-life disruption of normal physiology triggered by high oxygen exposures and pro-inflammatory conditions and introduce the application of MEx as immunomodulators and growth-promoting mediators for multisystem therapy.

## Introduction

The survival of premature infants born at extremely low birth weight (ELBW) has improved dramatically due to recent advances in neonatal care (Matthews et al., 2015). However, 40% of infants born before 28 weeks of gestational age (GA < 28) are at risk of one or more long-term complications, as they often require prolonged mechanical ventilation and exposure to high and variable oxygen tension (Younge et al., 2017). Preterm ELBW infants, even those who do not require supplemental oxygen, are exposed to “perceived” hyperoxia that often causes direct tissue damage and/or triggers maladaptive physiological responses. Such disruption not only impacts lung growth but can also lead to multiorgan dysfunction including cardiac, retinal, and neurological deficits as well as gastrointestinal and renal abnormalities (Weinberger et al., 2002; Johnson et al., 2011). Bronchopulmonary dysplasia (BPD) and other morbidities associated with prematurity, such as retinopathy of prematurity (ROP) and neurodevelopmental disabilities, share a common pathogenesis (i.e., inflammation and oxygen toxicity) not adequately addressed with current established therapies. Therapeutic advances based on new concepts and less invasive techniques are being currently tested to prevent BPD injury and the consequences of post-delivery respiratory care but do not target the underpinnings of perinatal diseases that accompany BPD. Moreover, the contribution of placental, microbial, and immune factors to the extrapulmonary sequelae of prematurity is just being recognized, and it is prompting the search for new therapeutic options (Collins et al., 2017).

Mesenchymal stem/stromal cell (MSC)-derived extracellular vesicles (MEx) have shown considerable anti-inflammatory and regenerative capacity in preclinical animal models, (Lee et al., 2012; Sdrimas and Kourembanas, 2014; Willis et al., 2018b), and a safety study of stem cell-derived extracellular vesicles in preterm neonates at high risk for BPD is currently ongoing (NCT03857841). The MEx advantage lies in their potential to modify immune, vascular, and parenchymal processes precipitated by preterm birth and exacerbated by oxygen fluctuations. This article will incorporate existing knowledge of the outcomes of prematurity and BPD as relating to MEx treatments in preclinical models of neonatal diseases, provide new information and perspective on the topic, and conclude by advancing the postulation that MEx can serve as a unitary therapeutic vector against systemic multi-organ pathologies of prematurity with common underlying triggers.

## Prematurity and Associated Organ Pathology

BPD is a major cause of morbidity and mortality in surviving extremely preterm infants. Its incidence has increased between 2009 and 2012 (Stoll et al., 2015). BPD pathogenesis is triggered by prenatal factors, postnatal mechanical trauma, and hyperoxia during mechanical ventilation and is exacerbated by other stressors, including infection, inflammation, and pulmonary volume overload. The pathology of BPD has evolved during the past 50 years, thanks to the introduction of surfactant therapy (Jobe, 1999) and improvements in prenatal and neonatal care. Infants born during the late canalicular and early saccular stage of normal lung development are likely to develop BPD pathology that includes a reduced number of alveoli and secondary septa. The large decrease in surface area is associated with reduced and dysmorphic pulmonary microvasculature, leading to increased vascular resistance and pulmonary hypertension (Mourani et al., 2015). Survivors with severe BPD also manifest a deterioration of lung function and a higher risk of developing chronic obstructive pulmonary disease later in life (Broström et al., 2010).

The pathologic processes driving abnormal lung growth affect the development of other systems as well. It is estimated that about 25–50% of surviving preterm infants suffer neurodevelopmental deficits such as cognitive deterioration (Larroque et al., 2008; Linsell et al., 2018), functional disability (Johnson et al., 2009), behavioral and psychological abnormalities that manifest later in life (Olsen et al., 2018), and, in a small proportion of cases, development of cerebral palsy and motor impairment (Jarjour, 2015).

An additional outcome of extreme preterm survival and supplemental oxygen is the occurrence of ROP (Silverman, 2004). Advanced ROP may lead to retinal detachment and significant visual impairment (Tin et al., 2001; Sommer et al., 2014). Although, clinically, ROP is considered a vascular disease precipitated by the premature exposure to high oxygen tension, there is evidence that neurons within the retina are also affected, causing a reduction in visual acuity, perception, and performance with increasing age (Leung et al., 2018).

Current therapies designed to treat and prevent BPD and other prematurity-related complications attempt to ameliorate acute stress reactions and rapidly improve organ function. An update on the current pharmacological therapies for BPD has been reported by Michael and colleagues (Michael et al., 2018). For example, the use of postnatal corticosteroids reduces inflammation and facilitates extubation in ventilator-dependent preterm infants (Doyle et al., 2006). Milder ventilation approaches, targeted oxygen saturation levels, growth factors, antioxidants, and other drug therapies are also being considered as less invasive alternatives for the prevention and treatment of BPD (Collins et al., 2017). Anti-vascular endothelial growth factor (VEGF) and erythropoietin therapies are used in the treatment of ROP and preterm brain injury, respectively (Patel et al., 2016; Younge et al., 2017). However, these interventions have limited efficacy as they only target isolated organs and stages of the disease. More importantly, they may contribute to the development of a “new” and “evolving” pathophysiology of prematurity in which the long-term outcomes are difficult to predict. Preterm birth is associated with arrested structural or functional development of key organs, causing impairment that will likely persist into adulthood. Thus, the development of new protective strategies requires an understanding of the molecular and cellular vulnerabilities of developing organs during increased oxygenation and concomitant inflammation with the goal of finding common pathogenic mechanisms amenable to therapeutic intervention.

## Organ Development and Oxygen Vulnerability

The transition from *in utero* environment to extrauterine life in the preterm born neonate triggers a sudden rise of lung and systemic oxygen tension, and supplemental oxygen application intensifies their vulnerability to injury. Postnatal development of several organ systems including the lung, brain, gastrointestinal tract, and lymphoid organs occurs during defined and particularly vulnerable stages in humans and rodents (Picut and Parker, 2017). Oxygen fluctuations during this stage fuel an inflammatory response and cause the arrested growth of parenchyma as well as dysregulated angiogenic and immune responses which can be regarded as having a common mechanistic origin.

### Lung

Contrary to what occurs in full-term human neonates that are born during the alveolar stage, rodents are born at a term during the saccular stage of lung development. From approximately postnatal (P) day 4–P21, the sacculae undergo thinning and reconstruction in a process termed alveolarization. Capillaries also grow superimposed to the process of alveolarization to create a network for blood supply and air gas exchange. The lungs of human preterm newborns experience an increase in arterial oxygen tension, even without supplemental oxygen, which leads to arrested alveolarization and vascularization. Preclinical studies have demonstrated that low antioxidant levels underlie the activation of transcription factors and pathways leading to endothelial and alveolar type II cell dysfunction and survival and the inactivation of surfactant (Stenmark and Abman, 2005). Exposure to hyperoxia also results in pulmonary accumulation of inflammatory cells and increased cytokine production (Ambalavanan et al., 2009).

### Brain

In humans, the period of rapid brain growth and therefore greater vulnerability occurs during the last 3 months of pregnancy and continues postnatally with outgrowth and reorganization of the central nervous system. Similarly, although rodent brain development is generally completed at birth, cell apoptosis, neuronal pruning, and cell migration are still occurring by P7 and not completed until P14, while myelination progresses beyond P21, the equivalent of a 2-year-old human (Semple et al., 2013). Moreover, blood vessels in the rodent brain develop in parallel with the cortical parenchyma, due in part to their association with microglia during the first week of postnatal development (Mondo et al., 2020). A major pathological feature of preterm brain injury and early postnatal exposure to toxic levels of oxygen is the presence of white matter lesions. Oligodendrocyte precursors are highly sensitive to oxygen fluctuations (Back and Miller, 2014), and hyperoxia induces their apoptosis and reduces their proliferation, causing white matter deficiencies that persist well beyond the juvenile age (Schmitz et al., 2011). In addition, other cellular changes including abnormal glial maturation and phenotype (Schmitz et al., 2011; Leaw et al., 2017), cell death (Truttmann et al., 2020), and disrupted cortical development and neuronal maturation have been described in preclinical and preterm brain injury (Fletcher et al., 2017; Fleiss et al., 2020).

### Retina

Hyperoxia also plays an initiating role in the pathogenesis of ROP in preterm infants and in experimental models of oxygen induced-retinopathy (OIR) (Vessey et al., 2011; Zin and Gole, 2013). Retinal vascularization in humans is completed at 36–40 weeks post-conceptual age. Preterm birth suddenly exposes the incompletely vascularized retina to hyperoxia, leading to vascular structural and functional compromise. Glial cells have been implicated in the genesis of retinal pathology in animal models of OIR, as they support the development of the vasculature and regulate cell apoptosis and neuronal metabolism and activation (Vessey et al., 2011; Shen et al., 2012). Müller cells express angiogenic growth factors such as VEGF and up-regulate the expression of glial fibrillary acidic protein (GFAP) as a hallmark of reactive gliosis in response to pathological oxygen tension. Microglia, like those in the brain, are generally considered immune cells, but they also contribute to the vascular and neuronal pathologic effects of oxygen-induced toxicity.

It is clear that perinatal oxidant stress, inflammation, and other toxic stimuli adversely impact the premature neonate cared for in modern neonatal intensive care units, and these result in acute and long-term injuries. The lack of regenerative therapies addressing the multiple organ pathologies occurring during this critical developmental stage highlights the need to consider a new, for the lack of a better term, “holistic” approach that can redirect normal development and enhance early organ restoration.

## Mex as a Regenerative Agent for Perinatal Pathologies

Therapies based on the MSC secretome have gained increased attention as promising candidates for a multifaceted approach, impacting multiple pathological aspects of premature infant disease. MSCs, as most cells, secrete a plethora of heterogenous extracellular vesicles (EVs) of diverse biogenetic origins. The molecular composition and associated bioactive cargo of each EV class reflect both their biogenesis as well as the specific stimulus triggering their formation. EV biogenesis and nomenclature have been extensively reviewed by Yeung et al. (2019). On a gross level, EVs are categorized into three subclasses: small EVs (sEVs) (∼30–150 nm in diameter) represent the class that includes the exosomes, i.e., EVs generated through the endosomal pathway; microvesicles (∼100 nm–1 μm in diameter) are generated through budding from the plasma membrane, and apoptotic bodies (>1 μm) are generated through decomposition of apoptotic blebs. These subclasses of vesicles, defined through their biogenesis and biophysical properties (such as size, density, and predominant protein markers), remain vaguely defined, especially in terms of functionality, due to limitations in EV isolation and the absence of universally accepted characterization methodologies. The International Society of Extracellular Vesicles spearheads the efforts to establish standardization in nomenclature, definitions, and methodology in this field (Lener et al., 2015; Théry et al., 2018; Witwer et al., 2019).

Although the mechanisms by which MEx induce their therapeutic effect are only partially understood, they appear to promote tissue restoration through the modulation of immune cell phenotype in the inflamed tissue microenvironment. Our group has demonstrated that MEx suppress the levels of proinflammatory cytokines (IL6, TNF-α) and modulate the expression of anti-inflammatory markers (Cd206, Arginase-1) in proinflammatory (M1-like, LPS + IFNγ) and pro-remodeling (M2-like, IL4 + IL13) macrophages, respectively (Willis et al., 2018a). In the neonatal lung, a polarized “M2-like” macrophage phenotype is critical for the process of alveolarization and normal lung development (Jones et al., 2013). Immune system homeostasis, disrupted by high oxygen levels in murine models of BPD, appears to be restored by MEx treatment.

Paramount in the pursuance of MEx-based therapy for premature infant disease is the uniformity in MEx preparations. This emphasizes the need of reproducibility in the parental MSC clone characteristics, and factors such as age, gender, and health status should be taken into consideration when selecting MSC donors (Willis et al., 2017). MEx produced by either human umbilical cord Wharton’s jelly or bone marrow MSCs have been used in preclinical studies, and they have been shown to be equally efficacious (Willis et al., 2018a).

Our group has extensively explored the role of MEx as vectors for lung-targeted therapy. MEx delivery suppressed pulmonary inflammation and the development of pulmonary hypertension and vascular remodeling in a model of hypoxia-induced lung injury (Lee et al., 2012) while preventing and reverting core features of pulmonary fibrosis, lung inflammation, and aberrant pulmonary morphology induced by bleomycin administration (Mansouri et al., 2019). Importantly, we have demonstrated a therapeutic effect of MEx in experimental BPD, ameliorating disrupted alveolarization and angiogenesis and restoring pulmonary function in adults. These actions were mostly mediated through the anti-inflammatory action of MEx on pulmonary macrophage number and phenotype (Willis et al., 2018a). Supporting results were also reported by other investigators in the field (Ahn et al., 2018; Braun et al., 2018; Chaubey et al., 2018).

MEx have been reported to restore myelination and functional outcome in experimental adult rodent models of demyelinating disease (Laso-García et al., 2018). Furthermore, MEx ameliorate white matter injury (Ophelders et al., 2016), inflammation, and gliosis (Drommelschmidt et al., 2017) and protect against impaired growth and altered cortical development from ischemic insults in models of neonatal preterm brain injury (Sisa et al., 2019). In addition, the therapeutic effect of MEx administration in ROP has been demonstrated in preclinical models. In OIR, MEx protect against retinal ischemia by preserving vascular flow and retinal thinning and enhancing functional recovery while decreasing inflammation and apoptosis. Importantly, these studies indicated that EVs are taken up by retinal neurons, retinal ganglion cells, and microglia (Moisseiev et al., 2017). Other investigators, using human placental amniotic membrane-derived MSCs, have also shown reduced pathological neovascularization in OIR (Kim et al., 2016). Collectively, MEx hold a great promise to potentially enhance the recovery of multiple organs and tissues. Their pleiotropic beneficial effect warrants their translation into clinical applications for ameliorating multifactorial pathologies, underlying not only BPD but also other prematurity-associated diseases.

**FIGURE 1:**
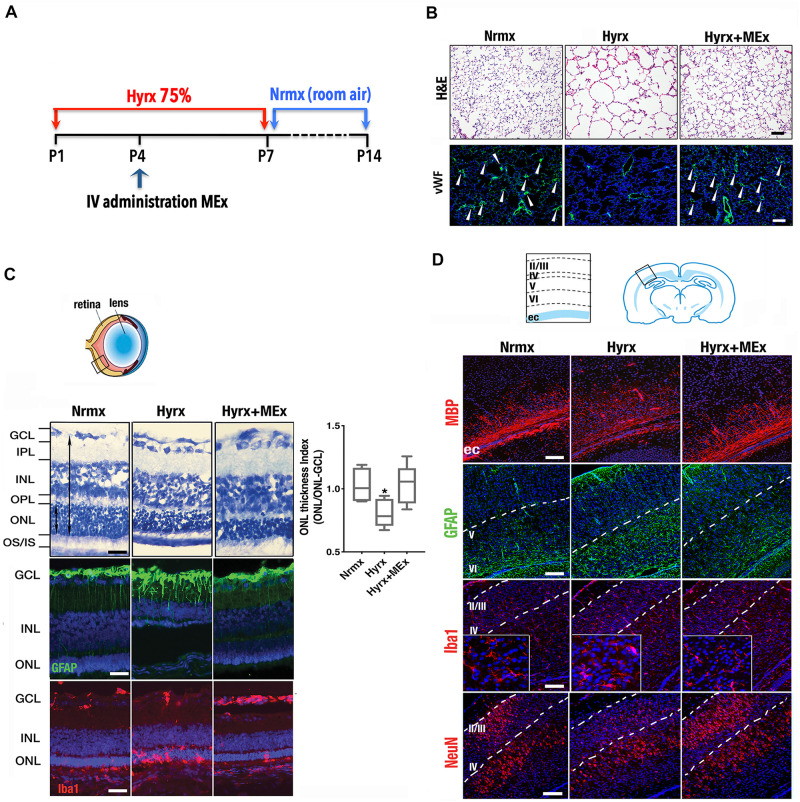
Mesenchymal stromal cell (MSC)-extracellular vesicle (MEx) treatment reduces systemic cellular and inflammatory responses. **(A)** Schematic showing the experimental design. Neonatal mice were exposed to hyperoxia (Hyrx; 75% O_2_) from postnatal day 1 (P1) to P7 and returned to room air (Nrmx) from P7 to P14 (this exposure modality impacts both alveolarization and myelination, as these processes progress postnatally). Hyrx mice were compared with age-matched control mice that remain in Nrmx conditions. The treated mice (Hyrx + MEx) received a single IV dose of MEx (50 μl corresponding to the product generated by 0.5 × 10^6^ MSCs injected *via* the superficial temporal vein) at P4 and were sacrificed immediately after Hyrx exposure (P7, period of maximal vulnerability for microglial activation and cortical migration) or after an additional 7 days in Nrmx (P14). MEx belong to a subset of “small” EVs that includes exosomes and were derived from either human umbilical cord Wharton’s jelly or bone marrow MSCs and purified in accordance with the 2018 Minimal Information for Studies of Extracellular Vesicles (Théry et al., 2018). Lungs, brains, and retinas were harvested, fixed, and processed for immunohistochemical and immunofluorescence analyses. **(B)** Disrupted alveolarization and vascularization in P14 Hyrx lungs was prevented with a single dose of MEx as shown in hematoxylin and eosin and von Willebrand factor-stained sections, respectively. Scale bar = 100 μm. **(C)** Hyperoxic injury of P14 retinal layers was prevented by MEx. Hyrx decreased retinal thickness as assessed with toluidine blue staining as a ratio of outer nuclear layer (ONL) thickness/ONL-ganglion cell layer (GCL) distance and shown to be restored with a single dose of MEx at P4 (Hyrx + MEx). Data are shown as mean ± SEM; *n* = 4 per group. *P < 0.05 vs. Nrmx (ANOVA followed by Tukey’s comparison test). Hyrx-induced gliosis at P14 is shown by GFAP immunofluorescence labeling of Müller cell bodies that are close to the inner nuclear layer with projections into the outer retina. MEx administration reverted Müller cell activation to Nrmx levels. Microglia activation and invasion into the ONL in P14 Hyrx retinas are depicted by increased ionized calcium binding adaptor molecule (Iba-1) immunofluorescence. MEx treatment restores microglial morphology and prevented invasion from injury. Scale bar = 25 μm. **(D)** Schematic depicting the cortical layers from which images were obtained. Myelin basic protein immunofluorescence showing marked staining decrease in the external capsule (ec) from P14 Hyrx mice and amelioration of white matter loss after a single dose of MEx at P4. Astrogliosis, as denoted by GFAP immunofluorescence staining at P14, was increased in early-exposed Hyrx mice but attenuated in the brains of MEx-treated littermates, particularly in areas in close association with white matter corresponding to the ec. Large Iba-1 positive cell bodies and thickened processes in P7 Hyrx cortex appear activated in comparison to Nrmx microglial cells. MEx normalized microglial morphological appearance in treated-Hyrx brain. Neuronal nuclear protein immunofluorescence staining of cortical P7 Hyrx brains, showing a less defined neuronal labeling pattern. MEx treatment restored cortical lamination after injury to Nrmx levels. Scale bar = 50 μm. GCL, ganglion cell layer; INL, inner nuclear layer; IPL, inner plexiform layer; IV, intravenous; ONL, outer nuclear layer; OS/IS, photoreceptor outer and inner segments junction; OPL, outer plexiform layer.

Considering that the pathobiology of the preterm newborn involves multiple organ systems that share common mechanisms (i.e., inflammation precipitated by oxygen toxicity), we considered it essential to explore the impact of systemic administration of MEx on postnatal lung, brain, and eyes using a model of multisystemic injury induced by hyperoxia exposure (see Figure 1A legend for section “Materials and Methods”). As demonstrated previously by our group (Willis et al., 2018a), hyperoxia (Hyrx) exposure to postnatal day 1 (P1) mice caused disrupted lung alveolarization and vascularization that were prevented with a single dose of MEx (Figure 1B). We now report that analysis of the retina in the same injured animals showed restoration of the retinal structure, decreased gliosis, and re-establishment of microglial homeostasis (Figure 1C) produced by Hyrx exposure, emphasizing the synergistic action of systemic MEx on immune and glial cell activation. In addition, white matter in the brain (Figure 1D) was preserved while astrogliosis, microglial response, and aberrant neuronal cortical pattern caused by Hyrx were reverted to normoxic (Nrmx) levels by MEx treatment, indicating a beneficial effect on myelination, gliosis, and neuronal density as the brain repairs and matures.

## The Regenerative Capacity of Mex Treatment for Postnatal Oxygen-Induced Multiple Organ System Injury: Perspectives From Newborn Medicine

**FIGURE 2:**
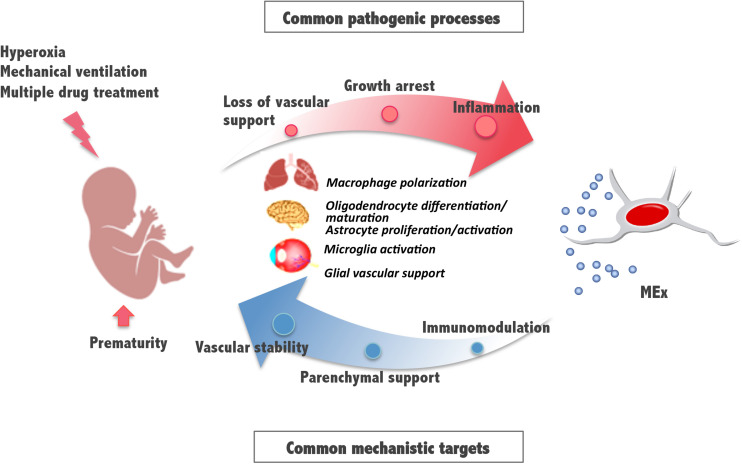
Schematic diagram illustrating mesenchymal stromal cell-extracellular vesicle (MEx) as an alternative systemic therapy for neonatal pathologies with common cellular targets. Prematurity and postnatal stressors can trigger pathological processes such as inflammation, growth arrest, and loss of vascular support, all of them essential to the function of the developing lung, brain, and retina. Multiple actions of MEx include modulation of macrophage and microglial cell activation and polarization, cell differentiation and maturation, and glial maintenance, all common mechanistic targets that support immunomodulation, preservation of parenchymal integrity, and vascular stability.

These and previously reported results indicate that, in addition to lung, the retina and brain, known to be also vulnerable to the detrimental effects of hyperoxia exposure, can benefit from an early systemic administration of MEx. Some pathologic mechanisms involve the activation of immune cells and supportive glial cells that are critical for the maintenance of the vasculature and tissue homeostasis and suggest common pathways and processes amenable for therapeutic intervention (Figure 2). Although the detailed molecular mechanisms of MEx action are still the focus of intensive investigation, the beneficial effects MEx bestow on perinatal pathologies seem to principally rely on their immunomodulatory actions on tissue-resident immune cells. Alveolar macrophages in the lung and microglia in the brain revert to their anti-inflammatory polarization state in response to MEx, favoring remodeling and tissue repair. In addition, MEx effects on brain and retina seem to suggest that, directly or indirectly through parenchymal glial cells, MEx promote normal vascular function and neuronal integrity. Although the major systemic action of MEx appears to be the modulation of inflammatory states precipitated by oxygen toxicity, we should note that their impact on myelination has been reported, in certain studies, to be associated with their ability to promote oligodendrocyte differentiation and maturation (Otero-Ortega et al., 2018).

Following early systemic administration, MEx can be delivered to injured developing tissues and improve organ development through anti-inflammatory, angiogenic, and pro-survival mechanisms. This early intervention would require the development of reliable biomarkers of disease and better brain and lung imaging tools, thus allowing the identification of patients likely to develop BPD and associated illnesses as early as possible. Most current investigations of live cell MSC-based or MEx-based therapeutic approaches are focused on evaluating the safety and efficacy of these treatments after preterm birth. However, efficient MEx therapies should also consider the effects on the host immune system. As preterm neonates are more skewed toward a tolerogenic development and are more susceptible to infections, the immunomodulatory impact of MEx treatment has to be considered when applying those therapies in compromised newborns with risk of infection. Efforts must be made to minimize contamination and aid consistency in MEx preparations. Finally, given the systemic effect of MEx, the advancement of *in vivo* tracing methods will help elucidate their interaction with target organs and help decipher the contribution of circulating EVs on systemic signaling and immunomodulation during the critical period surrounding preterm birth and the development of lung and multiorgan complications.

## Conclusion

This perspective advances the postulation that common triggers and pathways underlying dysregulated angiogenesis, growth, and immune system development converge in precipitating systemic neonatal injury in susceptible organs, including but not limited to the lung, brain, and retina. This demands new approaches to simultaneously address the potential pathogenic mechanisms of BPD and associated pathologies of prematurity. Numerous reports of preclinical studies, including those presented here, highlight the protective potential of MEx treatment against such early-life injuries. A phase I clinical study on the effects of MEx treatment in BPD is currently in progress, and basic research studies defining the molecular and cellular mechanisms responsible for the protective effects of MEx are actively underway. We are confident that future research in MEx therapies based on these strategies will lead to a more holistic approach for effectively preventing or treating complications of prematurity.

## Data Availability Statement

The raw data supporting the conclusions of this article will be made available by the authors, without undue reservation.

## Ethics Statement

The animal study was reviewed and approved by the Boston Children’s Hospital Animal Care and Use Committee.

## Author Contributions

AF-G and GW participated in study design and execution, data collection, and data analysis. VY and MR participated in study execution and data analysis. XL participated in study execution and technical assistance. SAM and SK contributed to study design, supervision of study execution, manuscript writing, data analysis, and final article editing and approval. All authors contributed to the article and approved the submitted version.

## Conflict of Interest

SAM and SK are named inventors on intellectual property licensed by Boston Children’s Hospital to United Therapeutics Corp. The remaining authors declare that the research was conducted in the absence of any commercial or financial relationships that could be construed as a potential conflict of interest.

## References

[B1] Ahn S. Y. Park W. S. Kim Y. E. Sung D. K. Sung S. I. Ahn J. Y. (2018). Vascular endothelial growth factor mediates the therapeutic efficacy of mesenchymal stem cell-derived extracellular vesicles against neonatal hyperoxic lung injury. *Exp. Mol. Med.* 50:26. 10.1038/s12276-018-0055-8 29650962 PMC5938045

[B2] Ambalavanan N. Carlo W. A. D’angio C. T. Mcdonald S. A. Das A. Schendel D. (2009). Cytokines associated with bronchopulmonary dysplasia or death in extremely low birth weight infants. *Pediatrics* 123 1132–1141. 10.1542/peds.2008-0526 19336372 PMC2903210

[B3] Back S. A. Miller S. P. (2014). Brain injury in premature neonates: a primary cerebral dysmaturation disorder? *Ann. Neurol.* 75 469–486. 10.1002/ana.24132 24615937 PMC5989572

[B4] Braun R. K. Chetty C. Balasubramaniam V. Centanni R. Haraldsdottir K. Hematti P. (2018). Intraperitoneal injection of MSC-derived exosomes prevent experimental bronchopulmonary dysplasia. *Biochem. Biophys. Res. Commun.* 503 2653–2658. 10.1016/j.bbrc.2018.08.019 30093115 PMC6398932

[B5] Broström E. B. Thunqvist P. Adenfelt G. Borling E. Katz-Salamon M. (2010). Obstructive lung disease in children with mild to severe BPD. *Respir. Med.* 104 362–370. 10.1016/j.rmed.2009.10.008 19906521

[B6] Chaubey S. Thueson S. Ponnalagu D. Alam M. A. Gheorghe C. P. Aghai Z. (2018). Early gestational mesenchymal stem cell secretome attenuates experimental bronchopulmonary dysplasia in part via exosome-associated factor TSG-6. *Stem Cell Res. Ther.* 9:173. 10.1186/s13287-018-0903-4 29941022 PMC6019224

[B7] Collins J. J. P. Tibboel D. De Kleer I. M. Reiss I. K. M. Rottier R. J. (2017). The future of bronchopulmonary dysplasia: emerging pathophysiological concepts and potential new avenues of treatment. *Front. Med. (Lausanne)* 4:61. 10.3389/fmed.2017.00061 28589122 PMC5439211

[B8] Doyle L. W. Davis P. G. Morley C. J. Mcphee A. Carlin J. B. (2006). Low-dose dexamethasone facilitates extubation among chronically ventilator-dependent infants: a multicenter, international, randomized, controlled trial. *Pediatrics* 117 75–83. 10.1542/peds.2004-2843 16396863

[B9] Drommelschmidt K. Serdar M. Bendix I. Herz J. Bertling F. Prager S. (2017). Mesenchymal stem cell-derived extracellular vesicles ameliorate inflammation-induced preterm brain injury. *Brain Behav. Immun.* 60 220–232. 10.1016/j.bbi.2016.11.011 27847282

[B10] Fleiss B. Gressens P. Stolp H. B. (2020). Cortical gray matter injury in encephalopathy of prematurity: link to neurodevelopmental disorders. *Front. Neurol.* 11:575. 10.3389/fneur.2020.00575 32765390 PMC7381224

[B11] Fletcher E. Wade J. Georgala P. A. Gillespie T. L. Price D. J. Pilley E. (2017). Oxygen flux reduces Cux1 positive neurons and cortical growth in a gestational rodent model of growth restriction. *Ann. Anat.* 210 84–93. 10.1016/j.aanat.2016.11.014 27986613

[B12] Jarjour I. T. (2015). Neurodevelopmental outcome after extreme prematurity: a review of the literature. *Pediatr. Neurol.* 52 143–152. 10.1016/j.pediatrneurol.2014.10.027 25497122

[B13] Jobe A. J. (1999). The new BPD: an arrest of lung development. *Pediatr. Res.* 46 641–643. 10.1203/00006450-199912000-00007 10590017

[B14] Johnson K. Scott S. D. Fraser K. D. (2011). Oxygen use for preterm infants: factors that may influence clinical decisions surrounding oxygen titration. *Adv. Neonatal Care* 11 8–14. quiz 15-6, 10.1097/ANC.0b013e318206d0c0 21285649

[B15] Johnson S. Fawke J. Hennessy E. Rowell V. Thomas S. Wolke D. (2009). Neurodevelopmental disability through 11 years of age in children born before 26 weeks of gestation. *Pediatrics* 124 e249–e257. 10.1542/peds.2008-3743 19651566

[B16] Jones C. V. Williams T. M. Walker K. A. Dickinson H. Sakkal S. Rumballe B. A. (2013). M2 macrophage polarisation is associated with alveolar formation during postnatal lung development. *Respir. Res.* 14:41. 10.1186/1465-9921-14-41 PMC3626876 23560845

[B17] Kim K. S. Park J. M. Kong T. Kim C. Bae S. H. Kim H. W. (2016). Retinal angiogenesis effects of Tgf-β1 and paracrine factors secreted from human placental stem cells in response to a pathological environment. *Cell Transplant.* 25 1145–1157. 10.3727/096368915X688263 26065854

[B18] Larroque B. Ancel P. Y. Marret S. Marchand L. André M. Arnaud C. (2008). Neurodevelopmental disabilities and special care of 5-year-old children born before 33 weeks of gestation (the EPIPAGE study): a longitudinal cohort study. *Lancet* 371 813–820. 10.1016/S0140-6736(08)60380-3 18328928

[B19] Laso-García F. Ramos-Cejudo J. Carrillo-Salinas F. J. Otero-Ortega L. Feliú A. Gómez-De Frutos M. (2018). Therapeutic potential of extracellular vesicles derived from human mesenchymal stem cells in a model of progressive multiple sclerosis. *PLoS One* 13:e0202590. 10.1371/journal.pone.0202590 30231069 PMC6145506

[B20] Leaw B. Zhu D. Tan J. Muljadi R. Saad M. I. Mockler J. C. (2017). Human amnion epithelial cells rescue cell death via immunomodulation of microglia in a mouse model of perinatal brain injury. *Stem Cell Res. Ther.* 8:46. 10.1186/s13287-017-0496-3 28241859 PMC5330154

[B21] Lee C. Mitsialis S. A. Aslam M. Vitali S. H. Vergadi E. Konstantinou G. (2012). Exosomes mediate the cytoprotective action of mesenchymal stromal cells on hypoxia-induced pulmonary hypertension. *Circulation* 126 2601–2611. 10.1161/CIRCULATIONAHA.112.114173 23114789 PMC3979353

[B22] Lener T. Gimona M. Aigner L. Börger V. Buzas E. Camussi G. (2015). Applying extracellular vesicles based therapeutics in clinical trials – an ISEV position paper. *J. Extracell. Vesicles* 4:30087. 10.3402/jev.v4.30087 26725829 PMC4698466

[B23] Leung M. P. Thompson B. Black J. Dai S. Alsweiler J. M. (2018). The effects of preterm birth on visual development. *Clin. Exp. Optom.* 101 4–12. 10.1111/cxo.12578 28868651

[B24] Linsell L. Johnson S. Wolke D. O’Reilly H. Morris J. K. Kurinczuk J. J. (2018). Cognitive trajectories from infancy to early adulthood following birth before 26 weeks of gestation: a prospective, population-based cohort study. *Arch. Dis. Child.* 103 363–370. 10.1136/archdischild-2017-313414 29146572 PMC5890637

[B25] Mansouri N. Willis G. R. Fernandez-Gonzalez A. Reis M. Nassiri S. Mitsialis S. A. (2019). Mesenchymal stromal cell exosomes prevent and revert experimental pulmonary fibrosis through modulation of monocyte phenotypes. *JCI Insight* 4:e128060. 10.1172/jci.insight.128060 31581150 PMC6948760

[B26] Matthews T. J. Macdorman M. F. Thoma M. E. (2015). Infant mortality statistics from the 2013 period linked birth/infant death data set. *Natl. Vital Stat. Rep.* 64 1–30. 26270610

[B27] Michael Z. Spyropoulos F. Ghanta S. Christou H. (2018). Bronchopulmonary dysplasia: an update of current pharmacologic therapies and new approaches. *Clin. Med. Insights Pediatr.* 12:1179556518817322. 10.1177/1179556518817322 30574005 PMC6295761

[B28] Moisseiev E. Anderson J. D. Oltjen S. Goswami M. Zawadzki R. J. Nolta J. A. (2017). Protective effect of intravitreal administration of exosomes derived from mesenchymal stem cells on retinal ischemia. *Curr. Eye Res.* 42 1358–1367. 10.1080/02713683.2017.1319491 28636406 PMC7008268

[B29] Mondo E. Becker S. C. Kautzman A. G. Schifferer M. Baer C. E. Chen J. (2020). A developmental analysis of juxtavascular microglia dynamics and interactions with the vasculature. *J. Neurosci.* 40 6503–6521. 10.1523/JNEUROSCI.3006-19.2020 32661024 PMC7486666

[B30] Mourani P. M. Sontag M. K. Younoszai A. Miller J. I. Kinsella J. P. Baker C. D. (2015). Early pulmonary vascular disease in preterm infants at risk for bronchopulmonary dysplasia. *Am. J. Respir. Crit. Care Med.* 191 87–95. 10.1164/rccm.201409-1594OC 25389562 PMC4299632

[B31] Olsen A. Dennis E. L. Evensen K. A. I. Husby Hollund I. M. Løhaugen G. C. C. Thompson P. M. (2018). Preterm birth leads to hyper-reactive cognitive control processing and poor white matter organization in adulthood. *Neuroimage* 167 419–428. 10.1016/j.neuroimage.2017.11.055 29191480 PMC6625518

[B32] Ophelders D. R. Wolfs T. G. Jellema R. K. Zwanenburg A. Andriessen P. Delhaas T. (2016). Mesenchymal stromal cell-derived extracellular vesicles protect the fetal brain after hypoxia-ischemia. *Stem Cells Transl. Med.* 5 754–763. 10.5966/sctm.2015-0197 27160705 PMC4878333

[B33] Otero-Ortega L. Gómez De Frutos M. C. Laso-García F. Rodríguez-Frutos B. Medina-Gutiérrez E. López J. A. (2018). Exosomes promote restoration after an experimental animal model of intracerebral hemorrhage. *J. Cereb. Blood Flow Metab.* 38 767–779. 10.1177/0271678X17708917 28524762 PMC5987932

[B34] Patel J. R. Ranjan S. S. Wasserman B. N. (2016). Antivascular endothelial growth factor in the treatment of retinopathy of prematurity. *Curr. Opin. Ophthalmol.* 27 387–392. 10.1097/ICU.0000000000000286 27206263

[B35] Picut C. A. Parker G. A. (2017). Postnatal organ development as a complicating factor in juvenile toxicity studies in rats. *Toxicol. Pathol.* 45 248–252. 10.1177/0192623316671609 27753635

[B36] Schmitz T. Ritter J. Mueller S. Felderhoff-Mueser U. Chew L. J. Gallo V. (2011). Cellular changes underlying hyperoxia-induced delay of white matter development. *J. Neurosci.* 31 4327–4344. 10.1523/JNEUROSCI.3942-10.2011 21411673 PMC3092487

[B37] Sdrimas K. Kourembanas S. (2014). MSC microvesicles for the treatment of lung disease: a new paradigm for cell-free therapy. *Antioxid. Redox Signal.* 21 1905–1915. 10.1089/ars.2013.5784 24382303 PMC4202925

[B38] Semple B. D. Blomgren K. Gimlin K. Ferriero D. M. Noble-Haeusslein L. J. (2013). Brain development in rodents and humans: identifying benchmarks of maturation and vulnerability to injury across species. *Prog. Neurobiol.* 10 1–16. 10.1016/j.pneurobio.2013.04.001 23583307 PMC3737272

[B39] Shen W. Fruttiger M. Zhu L. Chung S. H. Barnett N. L. Kirk J. K. (2012). Conditional Müllercell ablation causes independent neuronal and vascular pathologies in a novel transgenic model. *J. Neurosci.* 32 15715–15727. 10.1523/JNEUROSCI.2841-12.2012 23136411 PMC4014009

[B40] Silverman W. A. (2004). A cautionary tale about supplemental oxygen: the albatross of neonatal medicine. *Pediatrics* 113 394–396. 10.1542/peds.113.2.394 14754955

[B41] Sisa C. Kholia S. Naylor J. Herrera Sanchez M. B. Bruno S. Deregibus M. C. (2019). Mesenchymal stromal cell derived extracellular vesicles reduce hypoxia-ischaemia induced perinatal brain injury. *Front. Physiol.* 10:282. 10.3389/fphys.2019.00282 30941062 PMC6433879

[B42] Sommer A. Taylor H. R. Ravilla T. D. West S. Lietman T. M. Keenan J. D. (2014). Challenges of ophthalmic care in the developing world. *JAMA Ophthalmol.* 132 640–644. 10.1001/jamaophthalmol.2014.84 24604415 PMC4063878

[B43] Stenmark K. R. Abman S. H. (2005). Lung vascular development: implications for the pathogenesis of bronchopulmonary dysplasia. *Annu. Rev. Physiol.* 67 623–661. 10.1146/annurev.physiol.67.040403.102229 15709973

[B44] Stoll B. J. Hansen N. I. Bell E. F. Walsh M. C. Carlo W. A. Shankaran S. (2015). Trends in care practices, morbidity, and mortality of extremely preterm neonates, 1993-2012. *JAMA* 314 1039–1051. 10.1001/jama.2015.10244 26348753 PMC4787615

[B45] Théry C. Witwer K. W. Aikawa E. Alcaraz M. J. Anderson J. D. Andriantsitohaina R. (2018). Minimal information for studies of extracellular vesicles 2018 (MISEV2018): a position statement of the International society for extracellular vesicles and update of the MISEV2014 guidelines. *J. Extracell. Vesicles* 7:1535750. 10.1080/20013078.2018.1535750 30637094 PMC6322352

[B46] Tin W. Milligan D. W. Pennefather P. Hey E. (2001). Pulse oximetry, severe retinopathy, and outcome at one year in babies of less than 28 weeks gestation. *Arch. Dis. Child. Fetal Neonatal Ed.* 84 F106–F110. 10.1136/fn.84.2.F106 11207226 PMC1721225

[B47] Truttmann A. C. Ginet V. Puyal J. (2020). Current evidence on cell death in preterm brain injury in human and preclinical models. *Front. Cell Dev. Biol.* 8:27. 10.3389/fcell.2020.00027 32133356 PMC7039819

[B48] Vessey K. A. Wilkinson-Berka J. L. Fletcher E. L. (2011). Characterization of retinal function and glial cell response in a mouse model of oxygen-induced retinopathy. *J. Comp. Neurol.* 519 506–527. 10.1002/cne.22530 21192081

[B49] Weinberger B. Laskin D. L. Heck D. E. Laskin J. D. (2002). Oxygen toxicity in premature infants. *Toxicol. Appl. Pharmacol.* 181 60–67. 10.1006/taap.2002.9387 12030843

[B50] Willis G. R. Fernandez-Gonzalez A. Anastas J. Vitali S. H. Liu X. Ericsson M. (2018a). Mesenchymal stromal cell exosomes ameliorate experimental bronchopulmonary dysplasia and restore lung function through macrophage immunomodulation. *Am. J. Respir. Crit. Care Med.* 197 104–116. 10.1164/rccm.201705-0925OC 28853608 PMC5765387

[B51] Willis G. R. Kourembanas S. Mitsialis S. A. (2017). Toward Exosome-Based Therapeutics: isolation, Heterogeneity, and Fit-for-Purpose Potency. *Front. Cardiovasc. Med.* 4:63. 10.3389/fcvm.2017.00063 29062835 PMC5640880

[B52] Willis G. R. Mitsialis S. A. Kourembanas S. (2018b). “Good things come in small packages”: application of exosome-based therapeutics in neonatal lung injury. *Pediatr. Res.* 83 298–307. 10.1038/pr.2017.256 28985201 PMC5876073

[B53] Witwer K. W. Van Balkom B. W. M. Bruno S. Choo A. Dominici M. Gimona M. (2019). Defining mesenchymal stromal cell (MSC)-derived small extracellular vesicles for therapeutic applications. *J. Extracell. Vesicles* 8:1609206. 10.1080/20013078.2019.1609206 PMC6493293 31069028

[B54] Yeung V. Willis G. R. Taglauer E. Mitsialis S. A. Kourembanas S. (2019). “Paving the road for mesenchymal stem cell-derived exosome therapy in bronchopulmonary dysplasia and pulmonary hypertension,” in *Stem Cell-Based Therapy for Lung Disease* (Cham: Springer International Publishing), 131–152.

[B55] Younge N. Goldstein R. F. Bann C. M. Hintz S. R. Patel R. M. Smith P. B. (2017). Survival and neurodevelopmental outcomes among periviable infants. *N.Engl. J. Med.* 376 617–628. 10.1056/NEJMoa1605566 28199816 PMC5456289

[B56] Zin A. Gole G. A. (2013). Retinopathy of prematurity-incidence today. *Clin. Perinatol.* 40 185–200. 10.1016/j.clp.2013.02.001 23719304

